# Supplementary figures and images for: Comparative analyses of chloroplast genomes in ‘Red Fuji’ apples: low rate of chloroplast genome mutations
Source: PeerJ. 2022 Feb 21;10:e12927. doi: 10.7717/peerj.12927 (PMC8868015; doi:10.7717/peerj.12927)

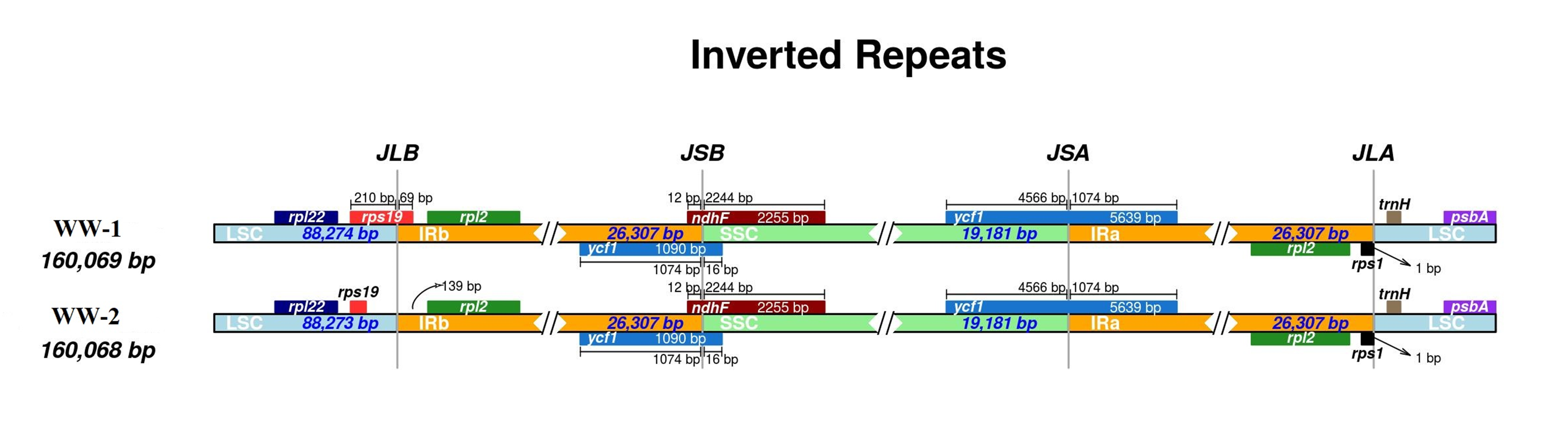

Supplement: Supplemental Information 1 [file peerj-10-12927-s001.png]

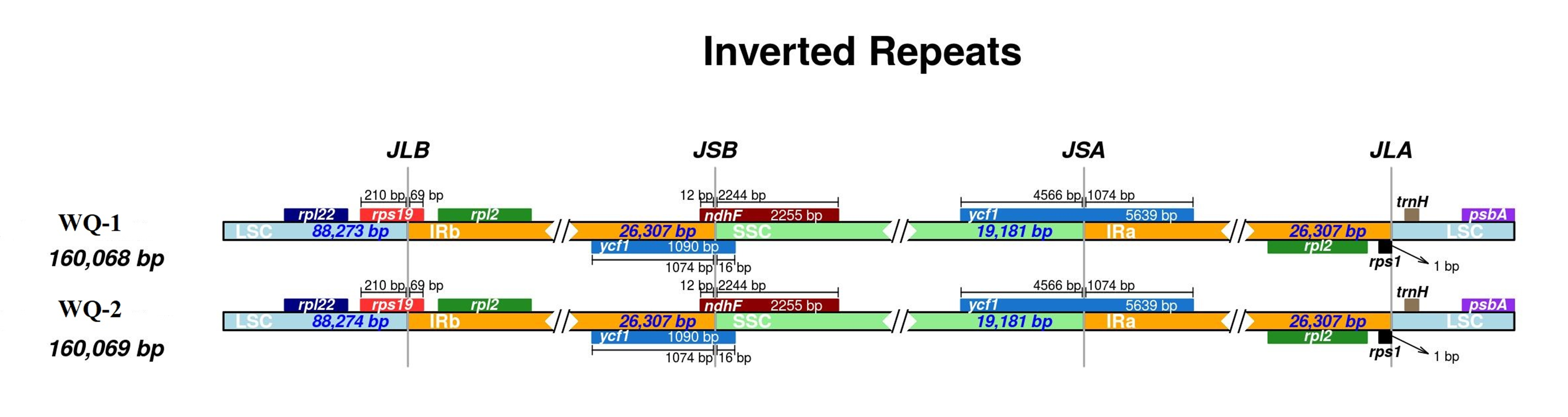

Supplement: Supplemental Information 2 [file peerj-10-12927-s002.png]

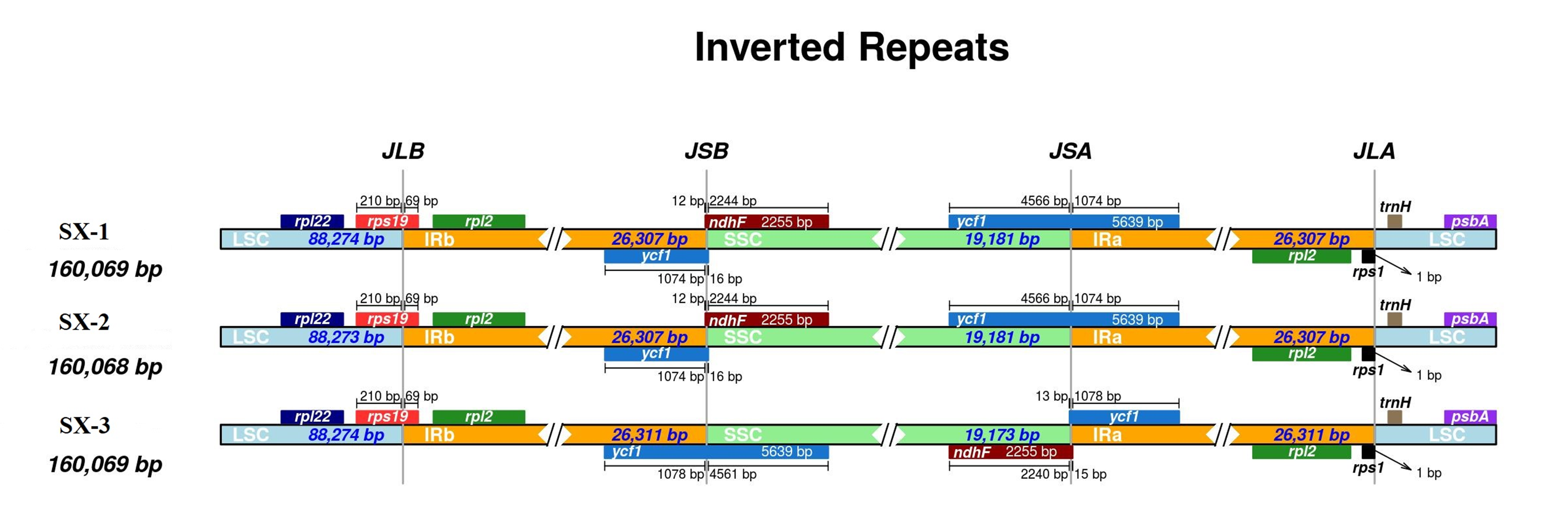

Supplement: Supplemental Information 3 [file peerj-10-12927-s003.png]

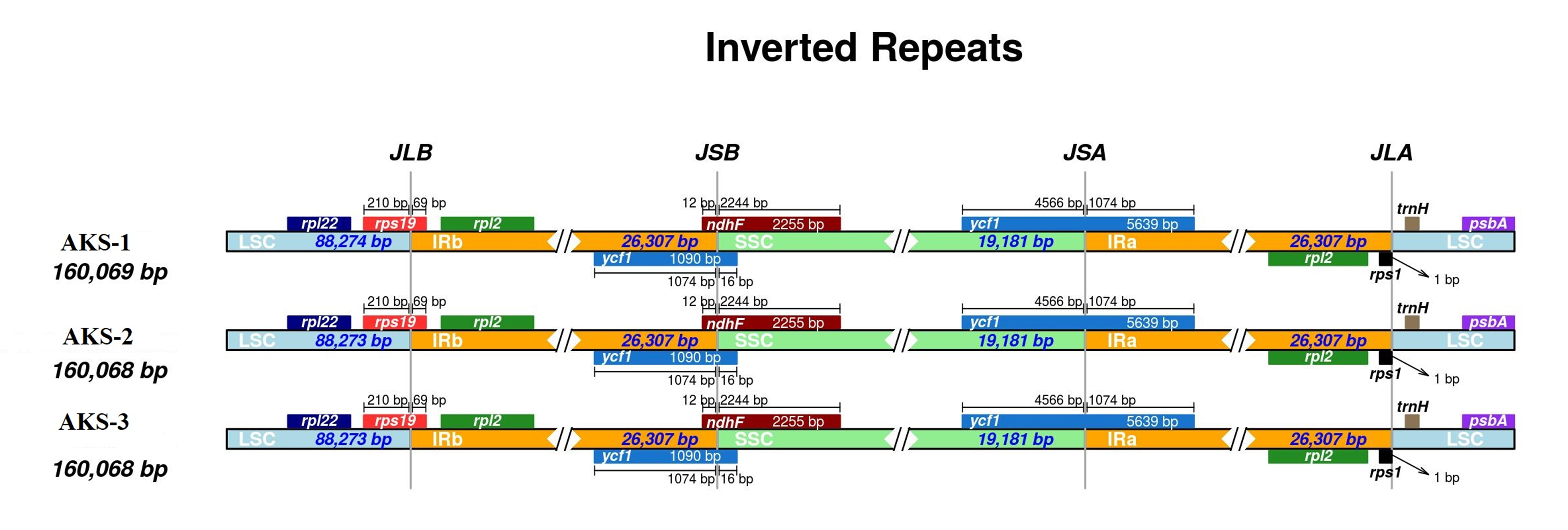

Supplement: Supplemental Information 4 [file peerj-10-12927-s004.png]

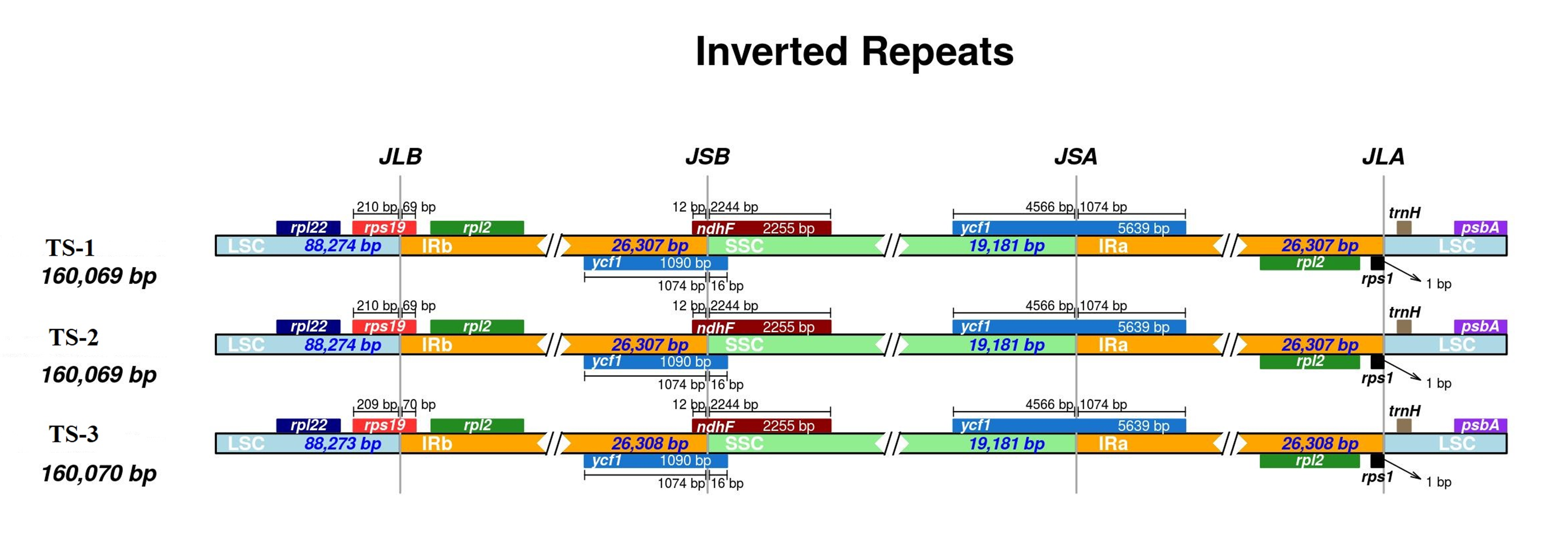

Supplement: Supplemental Information 5 [file peerj-10-12927-s005.png]

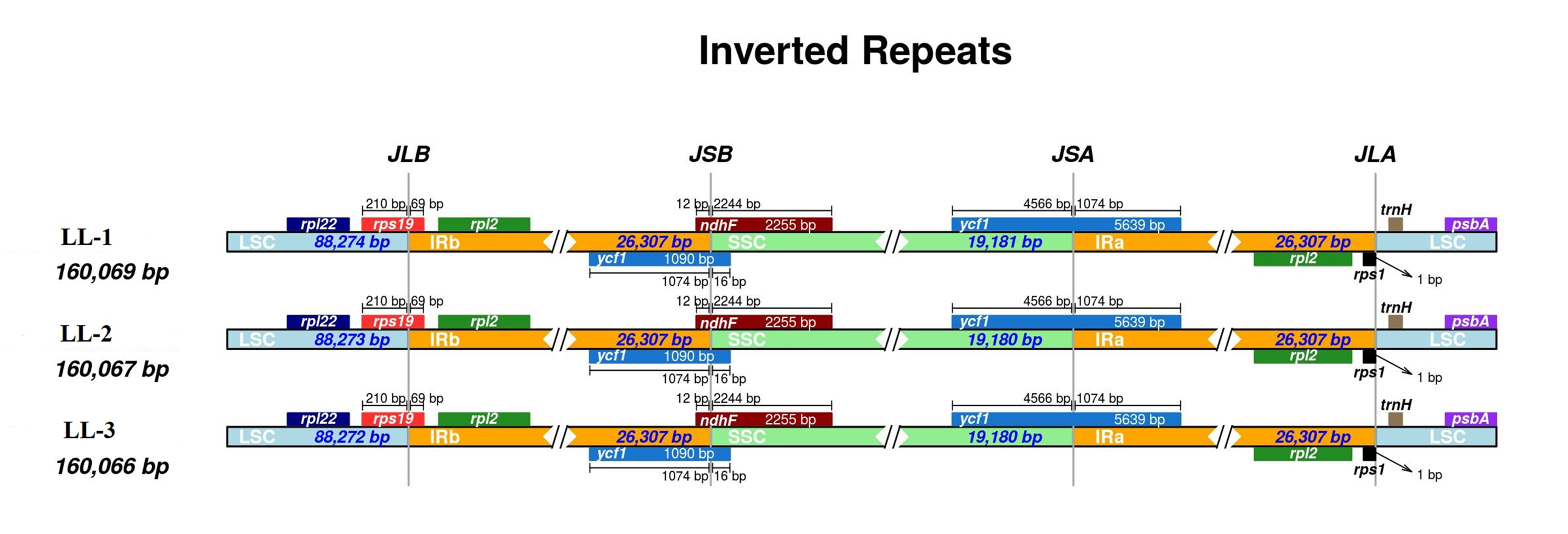

Supplement: Supplemental Information 6 [file peerj-10-12927-s006.png]

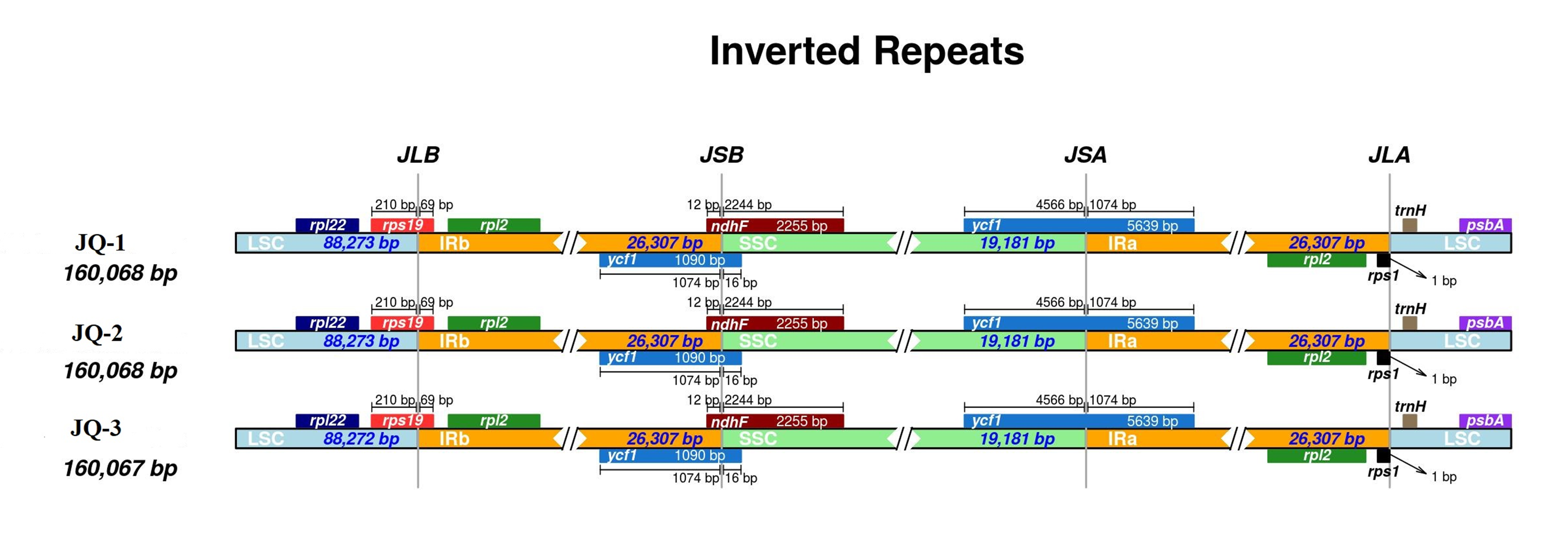

Supplement: Supplemental Information 7 [file peerj-10-12927-s007.png]

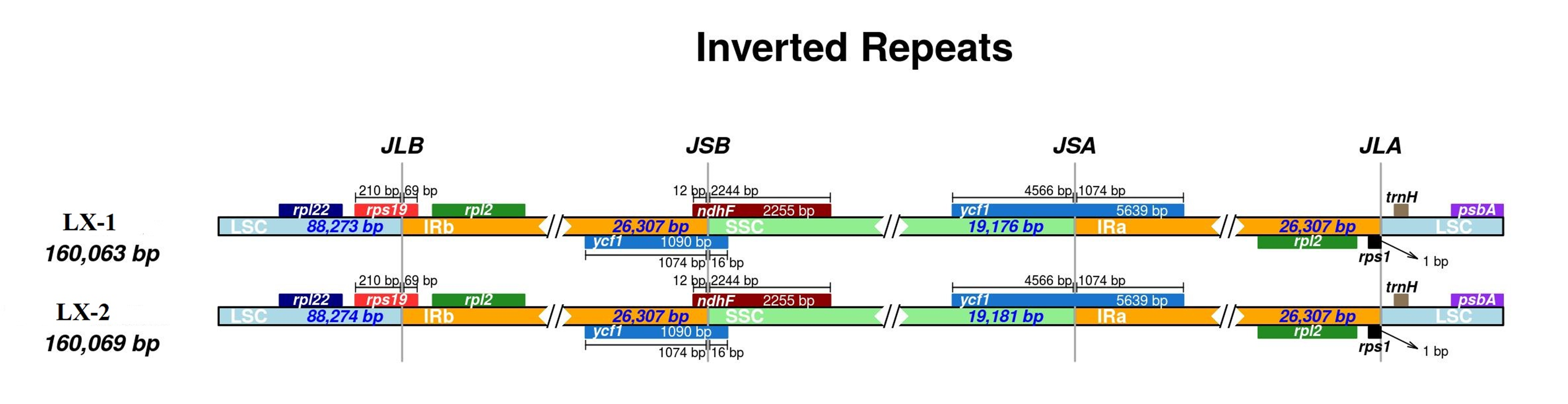

Supplement: Supplemental Information 8 [file peerj-10-12927-s008.png]

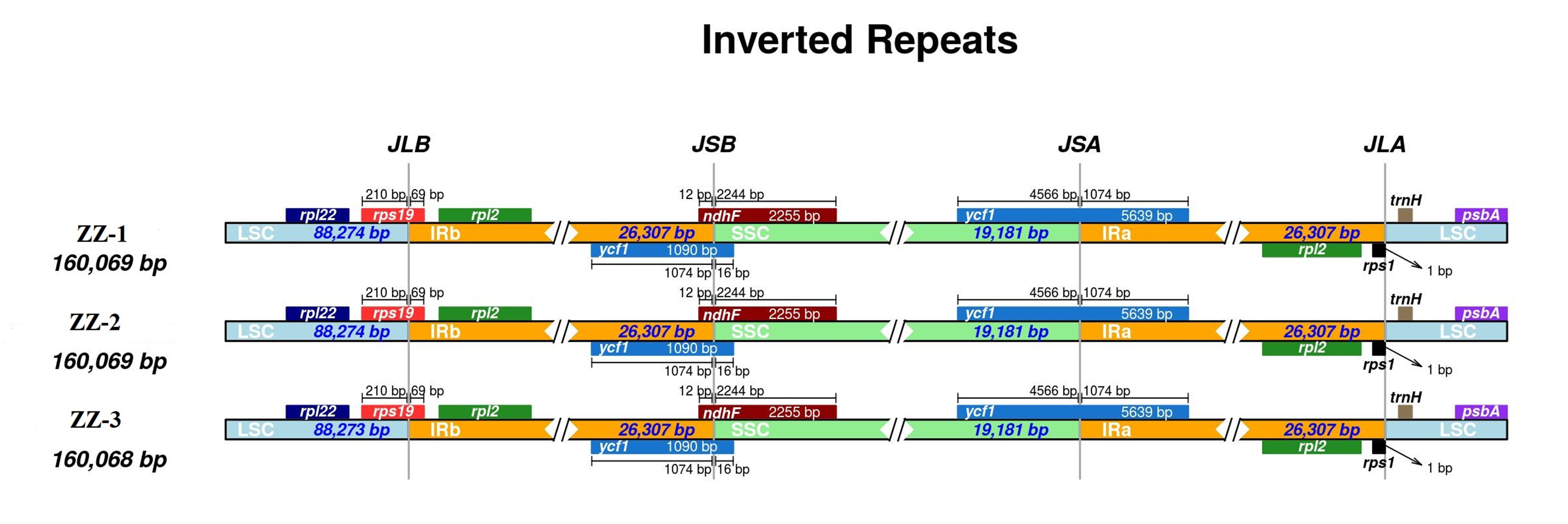

Supplement: Supplemental Information 9 [file peerj-10-12927-s009.png]

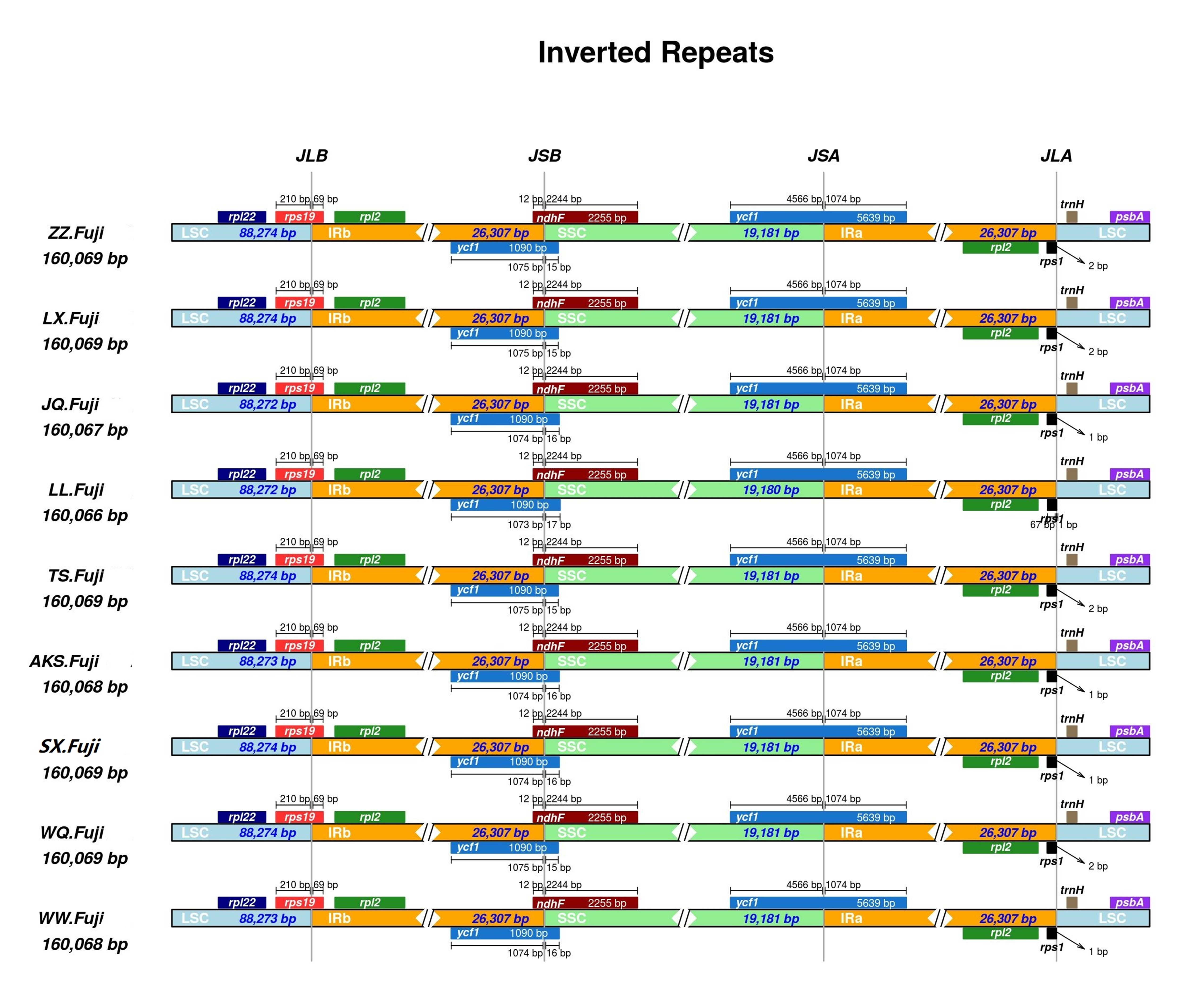

Supplement: Supplemental Information 10 [file peerj-10-12927-s010.png]
