# Supplementary material for: Comparative analyses of chloroplast genomes in ‘Red Fuji’ apples: low rate of chloroplast genome mutations
Source: PeerJ. 2022 Feb 21;10:e12927. doi: 10.7717/peerj.12927 (PMC8868015; doi:10.7717/peerj.12927)
Supplement: Supplemental Information 11 [file peerj-10-12927-s011.docx]

| Sample | Raw Base(bp) | Clean Base(bp) | Effective Rate(%) | Error Rate(%) | Q20(%) | Q30(%) | GC Content(%) |
| --- | --- | --- | --- | --- | --- | --- | --- |
| WW-1 | 28734623400 | 28695543900 | 99.86 | 0.03 | 96.98 | 91.81 | 38.55 |
| WW-2 | 18713730600 | 18680958000 | 99.82 | 0.03 | 96.67 | 91.28 | 39.82 |
| WQ-1 | 34673353500 | 34624084200 | 99.86 | 0.03 | 96.8 | 91.5 | 39.66 |
| WQ-2 | 20380603800 | 20359790400 | 99.9 | 0.03 | 95.74 | 89.08 | 39.05 |
| SX-1 | 22477735200 | 22452494100 | 99.89 | 0.03 | 96.64 | 91.16 | 39.03 |
| SX-2 | 19386949500 | 19363695000 | 99.88 | 0.03 | 96.13 | 94.29 | 39.16 |
| SX-3 | 15588082800 | 15567872700 | 99.87 | 0.03 | 96.85 | 91.53 | 38.72 |
| AKS-1 | 20168782800 | 20122320000 | 99.77 | 0.03 | 96.17 | 94.34 | 39.19 |
| AKS-2 | 16292287200 | 16271226300 | 99.87 | 0.03 | 96.79 | 91.47 | 38.83 |
| AKS-3 | 19701272700 | 19675436700 | 99.87 | 0.03 | 96.53 | 90.91 | 38.89 |
| TS-1 | 19264599000 | 19236433800 | 99.85 | 0.03 | 96.85 | 91.65 | 38.92 |
| TS-2 | 18793831200 | 18765515400 | 99.85 | 0.03 | 95.81 | 93.86 | 38.66 |
| TS-3 | 19894009200 | 19862538900 | 99.84 | 0.03 | 96.09 | 94.24 | 38.77 |
| LL-1 | 15351317700 | 15333168000 | 99.88 | 0.03 | 96.7 | 91.25 | 38.97 |
| LL-2 | 17151889500 | 17124161100 | 99.84 | 0.03 | 96.32 | 94.56 | 38.83 |
| LL-3 | 18327564600 | 18303221700 | 99.87 | 0.03 | 96.16 | 94.31 | 38.97 |
| JQ-1 | 18808694100 | 18786386400 | 99.88 | 0.03 | 97.73 | 93.5 | 38.5 |
| JQ-2 | 25067333400 | 25036309500 | 99.88 | 0.03 | 96.16 | 94.33 | 38.73 |
| JQ-3 | 18420029700 | 18395833800 | 99.87 | 0.03 | 96.36 | 94.62 | 38.86 |
| LX-1 | 19194286500 | 19166658300 | 99.86 | 0.03 | 96.31 | 94.54 | 38.77 |
| LX-2 | 19372944600 | 19337789100 | 99.82 | 0.03 | 96.09 | 94.26 | 38.75 |
| ZZ-1 | 22113265200 | 22072179300 | 99.81 | 0.03 | 95.94 | 94.04 | 38.8 |
| ZZ-2 | 31380203700 | 31318976400 | 99.8 | 0.03 | 96.15 | 89.94 | 39.24 |
| ZZ-3 | 25181831700 | 25136431200 | 99.82 | 0.03 | 96.12 | 94.28 | 38.97 |
