# Supplementary material for: Comparative analyses of chloroplast genomes in ‘Red Fuji’ apples: low rate of chloroplast genome mutations
Source: PeerJ. 2022 Feb 21;10:e12927. doi: 10.7717/peerj.12927 (PMC8868015; doi:10.7717/peerj.12927)
Supplement: Supplemental Information 12 [file peerj-10-12927-s012.docx]

| NO. | Name | GenBank numbers |
| --- | --- | --- |
| 1 | *Pyrus_pyrifolia* | MK172841.1 |
| 2 | *Pyrus_ussuriensis* | NC015996.1 |
| 3 | *Malus_sylvestrisA59* | MK434924 |
| 4 | *Malus_sieversii* | MK434920 |
| 5 | *Malus_baccata* | KX499859.1 |
| 6 | *Malus_micromalus* | MF062434.1 |
| 7 | *Malus_prunifolia* | KU851961.1 |
| 8 | *Malus_hupehensis* | NC040170.1 |
