# Supplementary material for: Comparative analyses of chloroplast genomes in ‘Red Fuji’ apples: low rate of chloroplast genome mutations
Source: PeerJ. 2022 Feb 21;10:e12927. doi: 10.7717/peerj.12927 (PMC8868015; doi:10.7717/peerj.12927)
Supplement: Supplemental Information 13 [file peerj-10-12927-s013.docx]

| NO. | Size(bp) | type | Repeat1start | Repeat2 start |
| --- | --- | --- | --- | --- |
| 1 | 65 | **F** | 39230 | 39257 |
| 2 | 63 | **P** | 55991 | 55991 |
| 3 | 45 | **P** | 0 | 88228 |
| 4 | 44 | **P** | 78193 | 78193 |
| 5 | 48 | **P** | 10252 | 10252 |
| 6 | 40 | **F** | 35102 | 35142 |
| 7 | 40 | **F** | 102903 | 125569 |
| 8 | 38 | **F** | 39230 | 39284 |
| 9 | 39 | **F** | 46758 | 102905 |
| 10 | 40 | **P** | 125569 | 145398 |
| 11 | 39 | **F** | 46758 | 145397 |
| 12 | 38 | **F** | 46758 | 125571 |
| 13 | 30 | **P** | 8352 | 48220 |
| 14 | 34 | **F** | 95582 | 95600 |
| 15 | 34 | **P** | 95582 | 152707 |
| 16 | 34 | **P** | 95600 | 152725 |
| 17 | 34 | **F** | 152707 | 152725 |
| 18 | 24 | **F** | 73720 | 73743 |
| 19 | 36 | **F** | 54798 | 54822 |
| 20 | 33 | **F** | 111858 | 111889 |
| 21 | 33 | **P** | 111858 | 136419 |
| 22 | 33 | **P** | 111889 | 136450 |
| 23 | 33 | **F** | 136419 | 136450 |
| 24 | 32 | **R** | 70994 | 70994 |
| 25 | 24 | **P** | 52449 | 83444 |
| 26 | 24 | **R** | 54843 | 54843 |
| 27 | 24 | **F** | 69435 | 69453 |
| 28 | 33 | **P** | 14001 | 14001 |
| 29 | 30 | **F** | 10339 | 10363 |
| 30 | 30 | **F** | 10471 | 10488 |
| 24 | 30 | **R** | 10618 | 10618 |
| 32 | 30 | **R** | 83445 | 83445 |
| 33 | 30 | **P** | 114115 | 114115 |
| 34 | 30 | **F** | 114115 | 134196 |
| 35 | 30 | **F** | 125998 | 126000 |
| 36 | 30 | **P** | 134196 | 134196 |
| 37 | 32 | **F** | 39457 | 39481 |
| 38 | 32 | **F** | 41779 | 44003 |
| 39 | 32 | **F** | 92460 | 92481 |
| 40 | 32 | **P** | 92460 | 155128 |
| 41 | 32 | **P** | 92481 | 155149 |
| 42 | 32 | **F** | 155128 | 155149 |
| 43 | 24 | **F** | 8351 | 38401 |
| 44 | 24 | **F** | 10464 | 50529 |
| 45 | 24 | **C** | 116816 | 125992 |
| 46 | 30 | **F** | 9886 | 9907 |
| 47 | 30 | **C** | 10260 | 34459 |
| 48 | 30 | **F** | 10412 | 10436 |
| 49 | 24 | **F** | 10520 | 10560 |

| NO. | Size(bp) | type | Repeat1start | Repeat2 start | location | | |  |
| --- | --- | --- | --- | --- | --- | --- | --- | --- |
| 1 |  |  |  |  |  |  |  | |
| 2 | 65 | 39231 | F | 65 | 39258 | 0 | 5.29e-30 | |
| 3 | 63 | 55992 | P | 63 | 55992 | -1 | 1.60e-26 | |
|  | 45 | 0 | P | 45 | 88229 | 0 | 5.82e-18 | |
|  | 44 | 78194 | P | 44 | 78194 | 0 | 2.33e-17 | |
|  | 48 | 10253 | P | 48 | 10253 | -2 | 9.23e-16 | |
|  | 40 | 35103 | F | 40 | 35143 | 0 | 5.96e-15 | |
|  | 40 | 102904 | F | 40 | 125570 | 0 | 5.96e-15 | |
|  | 40 | 125570 | P | 40 | 145399 | 0 | 5.96e-15 | |
|  | 38 | 39231 | F | 38 | 39285 | 0 | 9.54e-14 | |
|  | 39 | 46759 | F | 39 | 102906 | -1 | 2.79e-12 | |
|  | 39 | 46759 | P | 39 | 145398 | -1 | 2.79e-12 | |
|  | 38 | 46759 | F | 38 | 125572 | -1 | 1.09e-11 | |
|  | 30 | 8353 | P | 30 | 48221 | 0 | 6.25e-09 | |
|  | 34 | 95583 | F | 34 | 95601 | -2 | 1.23e-07 | |
|  | 34 | 95583 | P | 34 | 152708 | -2 | 1.23e-07 | |
|  | 34 | 95601 | P | 34 | 152726 | -2 | 1.23e-07 | |
|  | 34 | 152708 | F | 34 | 152726 | -2 | 1.23e-07 | |
|  | 31 | 73721 | F | 31 | 73744 | -1 | 1.45e-07 | |
|  | 36 | 54799 | F | 36 | 54823 | -3 | 2.94e-07 | |
|  | 33 | 111859 | F | 33 | 111890 | -2 | 4.64e-07 | |
|  | 33 | 111859 | P | 33 | 136420 | -2 | 4.64e-07 | |
|  | 33 | 111890 | P | 33 | 136451 | -2 | 4.64e-07 | |
|  | 33 | 136420 | F | 33 | 136451 | -2 | 4.64e-07 | |
|  | 32 | 70995 | R | 32 | 70995 | -2 | 1.74e-06 | |
|  | 31 | 52450 | P | 31 | 83445 | -2 | 6.54e-06 | |
|  | 31 | 54844 | R | 31 | 54844 | -2 | 6.54e-06 | |
|  | 31 | 69436 | F | 31 | 69454 | -2 | 6.54e-06 | |
|  | 33 | 14002 | P | 33 | 14002 | -3 | 1.44e-05 | |
|  | 30 | 10340 | F | 30 | 10364 | -2 | 2.45e-05 | |
|  | 30 | 10472 | F | 30 | 10489 | -2 | 2.45e-05 | |
|  | 30 | 10619 | R | 30 | 10619 | -2 | 2.45e-05 | |
|  | 30 | 83446 | R | 30 | 83446 | -2 | 2.45e-05 | |
|  | 30 | 114116 | P | 30 | 114116 | -2 | 2.45e-05 | |
|  | 30 | 114116 | F | 30 | 134197 | -2 | 2.45e-05 | |
|  | 30 | 125999 | F | 30 | 126001 | -2 | 2.45e-05 | |
|  | 30 | 134197 | P | 30 | 134197 | -2 | 2.45e-05 | |
|  | 32 | 39458 | F | 32 | 39482 | -3 | 5.23e-05 | |
|  | 32 | 41780 | F | 32 | 44004 | -3 | 5.23e-05 | |
|  | 32 | 93161 | F | 32 | 93182 | -3 | 5.23e-05 | |
|  | 32 | 93161 | P | 32 | 155129 | -3 | 5.23e-05 | |
|  | 32 | 93182 | P | 32 | 155150 | -3 | 5.23e-05 | |
|  | 32 | 155129 | F | 32 | 155150 | -3 | 5.23e-05 | |
|  | 31 | 8352 | F | 31 | 38402 | -3 | 1.90e-04 | |
|  | 31 | 10465 | F | 31 | 50530 | -3 | 1.90e-04 | |
|  | 31 | 10521 | F | 31 | 10561 | -3 | 1.90e-04 | |
|  | 31 | 116817 | C | 31 | 125993 | -3 | 1.90e-04 | |
|  | 30 | 9887 | F | 30 | 9908 | -3 | 6.85e-04 | |
|  | 30 | 10261 | C | 30 | 34460 | -3 | 6.85e-04 | |
|  | 30 | 10413 | F | 30 | 10437 | -3 | 6.85e-04 | |
